# A retrospective study on newborn screening for metabolic disorders

**DOI:** 10.6026/973206300181122

**Published:** 2022-12-31

**Authors:** Karam Chandrajit Singh, Prabhkiran Dhillon, Thushara Thulaseedharan

**Affiliations:** 1 Department of Obstretics and Gynaecologist, 7 Airforce Hospital, Kanpur-Cantt, Kanpur, Uttar Pradesh, India

**Keywords:** Newborn screening, G6PD deficiency, thyroid stimulating hormone, immunoreactive trypsinogen, profound biotinidase

## Abstract

The process of testing newborn infants for hormonal, genetic, metabolic, and other disorders is known as newborn screening (NSB). Newborn screening is essential for detecting, diagnosing, and treating disorders that could save serious consequences for a newborn's health. Congenital Hypothyroidism (CH), Cystic Fibrosis (CF), Glucose-6-phosphate dehydrogenase (G6PD) deficiency, and Profound Biotinidase deficiency (BD) are common disorders in India. A retrospective analysis of the results of NBS by Cord blood spots was performed at the department of Obstetrics and Gynecology, 7 Airforce Hospital, Kanpur, Uttar Pradesh, from June 2022 to September 2022. During this period, 26 newborns were screened for four disorders, including CH, CF, G6PD deficiency, and BD. In this investigation, no cases of CH, CF, G6PD deficiency, or BD were found to be positive. The results of the current data provide a distinct opportunity to investigate the birth prevalence of inborn metabolic disorders in the area close to the city of Kanpur.

## Background:

Newborn screening is a broad public health prevention programme that has saved thousands of infants from physical disabilities or death through early detection and treatment [1, 2]. In many countries around the world, newborn screening (NBS) is now a mandatory practice that has become an integral part of newborn investigation. An enzymatic defect in a single pathway of intermediate metabolism results in a wide range of disorders known as inborn errors of metabolism [3]. Neonatal disorders are currently the leading cause of perinatal and neonatal mortality in India, accounting for 9.2% of all cases in urban areas [4]. A neonatal disorder affects one out of every 1,000 newborns in India [5]. Congenital hypothyroidism (CH) is a thyroid hormone deficiency that occurs at birth. The most frequent causes of thyroid hormone deficiency at birth are disorders of thyroid hormone biosynthesis or difficulties with thyroid gland development (dysgenesis). Approximately 1:2,000 to 1:4,000 newborns have congenital hypothyroidism [6]. Cystic fibrosis (CF) is an inherited disorder that causes serious harm to the digestive system, lungs, and other organs in the body. Glucose-6-phosphate dehydrogenase deficiency is the most common enzyme deficiency, affecting approximately 400 million people worldwide [7, 8]. The deficiency is inherited and affects an enzyme necessary for red blood cell formation. Biotinidase deficiency is a genetic disorder that causes the body to be unable to recycle vitamin biotin. The more severe form of the condition, profound biotinidase deficiency, can cause seizures, weak muscle tone (hypotonia), breathing problems, hearing and vision loss, movement and balance problems [9]. Individuals suffering from these disorders who are undiagnosed and untreated, particularly those who have suffered permanent damage, continue to be a financial burden on their families and society. As a result, an effective NBS programme is essential in our country, especially given that diagnostic tests for these disorders are inexpensive and treatments are simple with a good prognosis. The current study aimed to screen neonates for Congenital Hypothyroidism, Cystic Fibrosis, G6PD deficiency, and Profound Biotinidase deficiency to expedite diagnosis and subsequent treatment and to facilitate the prevention of adverse outcomes.

## Materials and Methods:

## Blood Sampling:

Cord blood spot samples from 26 newborns were collected in satisfactory condition at the department of Obstetrics and Gynecology, 7 Airforce Hospital, Kanpur-Cantt, Kanpur, Uttar Pradesh, India, with the necessary details including neonate sex and birth weight. The blood was collected at the time of the neonate's delivery.

## Informed Consent:

All procedures were carried out by the committee on human experimentation's ethical standards (institutional and national). Before enrolling their newborn in the study, parents were counseled on the benefits of NBS and informed consent was obtained for screening tests to be performed on their neonates' DBS.

## Screening Procedures:

Thyroid Stimulating Hormone (TSH), Human Immunoreactive Trypsinogen (IRT), Glucose 6 Phosphate Dehydrogenase (G6PD), and Profound Biotinidase (BTD) levels were semi-quantitatively measured in cord blood spot specimens using Fluorometric Enzyme Immunoassay according to kit manufacturer's instructions (Perkin Elmer). The concentration of TSH not more than 34µlU/mL in blood was categorized as normal. A blood IRT concentration of not more than 110µg/L was considered normal. The normal biological reference interval for G6PD enzyme activity was 1.45U/gm of haemoglobin, and any sample with measured activity below this cutoff was considered positive. The normal biological reference interval for BTD activity was not less than 40U.

## Results and Discussion:

In India, untrained medical personnel attend 10% of deliveries, and only one-third of babies receive a health check-up by trained health personnel within two days of birth [10]. There is a lack of human resources with expertise in childcare and infrastructure for managing sick babies, which, coupled with the above observations greatly increase the odds of missing affected babies. In India, health is a state subject, which means that the budget, human resources, logistics, and microplanning of any NBS programme must be decentralised and mapped for each state. Early detection and treatment of common metabolic and genetic disorders should be a part of neonatal care because it can help prevent intellectual and physical defects as well as life-threatening illnesses. Congenital Hypothyroidism, Cystic Fibrosis, Glucose 6 Phosphate Dehydrogenase deficiency, and Profound Biotinidase deficiency aimed at and have successfully resulted in early detection and intervention. Screening tests of these disorders and their reference range were shown in (Table 1-see PDF) Three (11.53%) of the neonates had low birth weights (< 2.5kg) according to their demographic features. There were 15 male infants and 11 female infants. According to a 2014 study, the most common disorders are CH, cystic fibrosis, G6PD, and biotinidase deficiency [11]. All the analytes are within the normal range as shown in figure 1a, b, c & d. Two neonates had G6PD deficiency values of 1.98 and 2.02, which are close to the cut off value of 1.45U/gm of haemoglobin shown in figure 1c. CH is a well-understood disorder that is simple to diagnose and treat. Financially, the return on investment is extremely high [12]. Cystic fibrosis cases are uncommon in the Indian population, with a prevalence of 1/43,321 to 1/100,323 [13]. The CF is thought to be extremely rare in the Indian subcontinent. Screening among Indian subcontinent immigrants to the United Kingdom and the United States indicated a CF prevalence estimated to be between 1/10,000 and 1/40,000 [14]. On screening 32,903 newborns, Bisoi et al. discovered a 14.7% prevalence of G6PD [15]. Wolf B did a global survey for BD and estimated the incidence to be 1:61067 populations, despite the fact that severe or profound disease is quite rare (1: 137401 populations) [16]. This retrospective analysis reveals that CH, CF, G6PD deficiency, and BD are prevalent inherited metabolic disorders that should be checked for in Indian hospitals.

## Conclusion:

Newborn screening (NSB) has become a valuable method for detecting inborn metabolic disorders. The World Health Organization has advised newborn screening to reduce the prevalence of disorders. However, the current state of NBS in Asian countries is not very good. The main problems in implementing this programme have been a lack of funding, a lack of public awareness, insufficient manpower, and inadequate support services. A proportion of infants may be saved from disability if they are diagnosed early. The current study presented numerous data concerning demographic results in Kanpur, but many more studies in this direction are needed to determine the exact burden of Inborn Errors of Metabolism solutions to deal with these issues.

## Limitations of Study:

The main limitation of the study is its small sample size of newborns from 7 Airforce Hospital, Kanpur, Uttar Pradesh, India.The findings of this study are likely to change if a broader investigation is conducted across all the hospitals in Kanpur.
